# Rainfall as a trigger of ecological cascade effects in an Australian groundwater ecosystem

**DOI:** 10.1038/s41598-021-83286-x

**Published:** 2021-02-12

**Authors:** Mattia Saccò, Alison J. Blyth, William F. Humphreys, Steven J. B. Cooper, Nicole E. White, Matthew Campbell, Mahsa Mousavi-Derazmahalleh, Quan Hua, Debashish Mazumder, Colin Smith, Christian Griebler, Kliti Grice

**Affiliations:** 1 WA-Organic Isotope Geochemistry Centre, The Institute for Geoscience Research, School of Earth and Planetary Sciences, Curtin University, Perth, WA 6102 Australia; 2 Trace and Environmental DNA (TrEnD) Laboratory, School of Molecular and Life Sciences, Curtin University, Perth, WA 6102 Australia; 3 School of Molecular and Life Sciences, Curtin University, Perth, WA 6102 Australia; 4 Collections and Research Centre, Western Australian Museum, Welshpool, WA 6986 Australia; 5 School of Biological Sciences, University of Western Australia, Crawley, WA 6009 Australia; 6 Australian Centre for Evolutionary Biology and Biodiversity, School of Biological Sciences, University of Adelaide, Adelaide, SA 5005 Australia; 7 Evolutionary Biology Unit, South Australian Museum, North Terrace, Adelaide, SA 5000 Australia; 8 Australian Nuclear Science and Technology Organisation (ANSTO), Locked Bag 2001, Kirrawee DC, NSW 2232 Australia; 9 Department of Archaeology and History, La Trobe University, Bundoora, VIC 3086 Australia; 10 Laboratorio de Evolución Humana, Departamento de Historia, Geografía y Comunicación, Universidad de Burgos, 09001 Burgos, Spain; 11 Department of Functional and Evolutionary Ecology, University of Vienna, 1090 Vienna, Austria

**Keywords:** Biochemistry, Ecology, Biogeochemistry, Ecosystem ecology, Freshwater ecology, Microbial ecology, Stable isotope analysis

## Abstract

Groundwaters host vital resources playing a key role in the near future. Subterranean fauna and microbes are crucial in regulating organic cycles in environments characterized by low energy and scarce carbon availability. However, our knowledge about the functioning of groundwater ecosystems is limited, despite being increasingly exposed to anthropic impacts and climate change-related processes. In this work we apply novel biochemical and genetic techniques to investigate the ecological dynamics of an Australian calcrete under two contrasting rainfall periods (LR—low rainfall and HR—high rainfall). Our results indicate that the microbial gut community of copepods and amphipods experienced a shift in taxonomic diversity and predicted organic functional metabolic pathways during HR. The HR regime triggered a cascade effect driven by microbes (OM processors) and exploited by copepods and amphipods (primary and secondary consumers), which was finally transferred to the aquatic beetles (top predators). Our findings highlight that rainfall triggers ecological shifts towards more deterministic dynamics, revealing a complex web of interactions in seemingly simple environmental settings. Here we show how a combined isotopic-molecular approach can untangle the mechanisms shaping a calcrete community. This design will help manage and preserve one of the most vital but underrated ecosystems worldwide.

## Introduction

Groundwaters, together with deep sea environments, are some of the least explored ecosystems in the world. Despite the recent upsurge in groundwater investigations, the subsurface ecological framework still suffers from a lack of knowledge, both in terms of their biological diversity and ecological functioning, notwithstanding groundwater’s environmental importance^1,2^.

Subsurface obligate aquatic fauna—namely stygofauna—display arrays of specific environmental characteristics (loss of eyes and pigmentation, long antennae, etc.)^3^. Stygofauna are perceived as adapted to a stable physico-chemical environment, and there is evidence of high degrees of resilience to the fluctuations of the environmental conditions, i.e. groundwater recharge, source of organic matter and energy^4^.

Rainfall events are considered major drivers in shaping hydrological dynamics in aquifers via processes like percolation or lateral flow^5^, and stygofauna respond both in function and community composition to these hydraulic shifts^6,7^. Several groundwater investigations indicate that inflows of terrestrial organic material (OM) cause ecological shifts within subsurface communities^8–10^. However, the interpretation of carbon flows and trophic web interactions within groundwater biota is far from straightforward^11^, with recent studies indicating composite pathways for the incorporation of OM in groundwaters^12,13^, and highlighting the need for interdisciplinary research that allows refinement of the subterranean ecological patterns^14^. Stable isotope chemistry (SIA—Stable Isotope Analysis, CSIA—Compound Specific Stable Isotope Analysis) and molecular biology (eDNA—environmental DNA, DNA metabarcoding, etc.) are two disciplines providing new perspectives in the study of ecological dynamics in freshwater environments^11^. However, these techniques are still mainly employed in marine and surface terrestrial environments, and their application in groundwaters is in its infancy^14^.

The arid western side of Australia, with its array of calcrete (carbonate) aquifers^15,16^ sustaining unique stygofaunal communities^17^, has been the focus of a large number of studies on taxonomy, biogeography and evolutionary patterns^18,19^. Numerous studies have focused on the Sturt Meadows calcrete^20,21^, including recent studies on microbial and stygofaunal ecological patterns across different rainfall periods indicating that the inflow of nutrients after rainfall triggers shifts in microbial metabolisms^22^, stygofaunal niche occupations^23^, and invertebrate trophic interactions^7^.

This study extends prior research by unravelling Sturt Meadows carbon end energy flows through isotopic (SIA, CSIA and ^14^C) and molecular investigations (DNA metabarcoding on bacteria from stygofaunal specimens). This investigation has three specific objectives: (1) unravel the biochemical paths that regulate the microbially-mediated nutrient assimilation among the stygofaunal community, (2) elucidate the flow of carbon and energy fluxes among primary/secondary consumers and predators and (3) understand the ecological functioning of the calcrete biotic community under two contrasting rainfall periods. We hypothesise that rainfall-driven biogeochemical dynamics play a key role in shaping the mechanisms regulating nutrient flows and food web interactions amongst the Sturt Meadows subterranean biota.

## Methodology

### Study area

**Figure 1:**
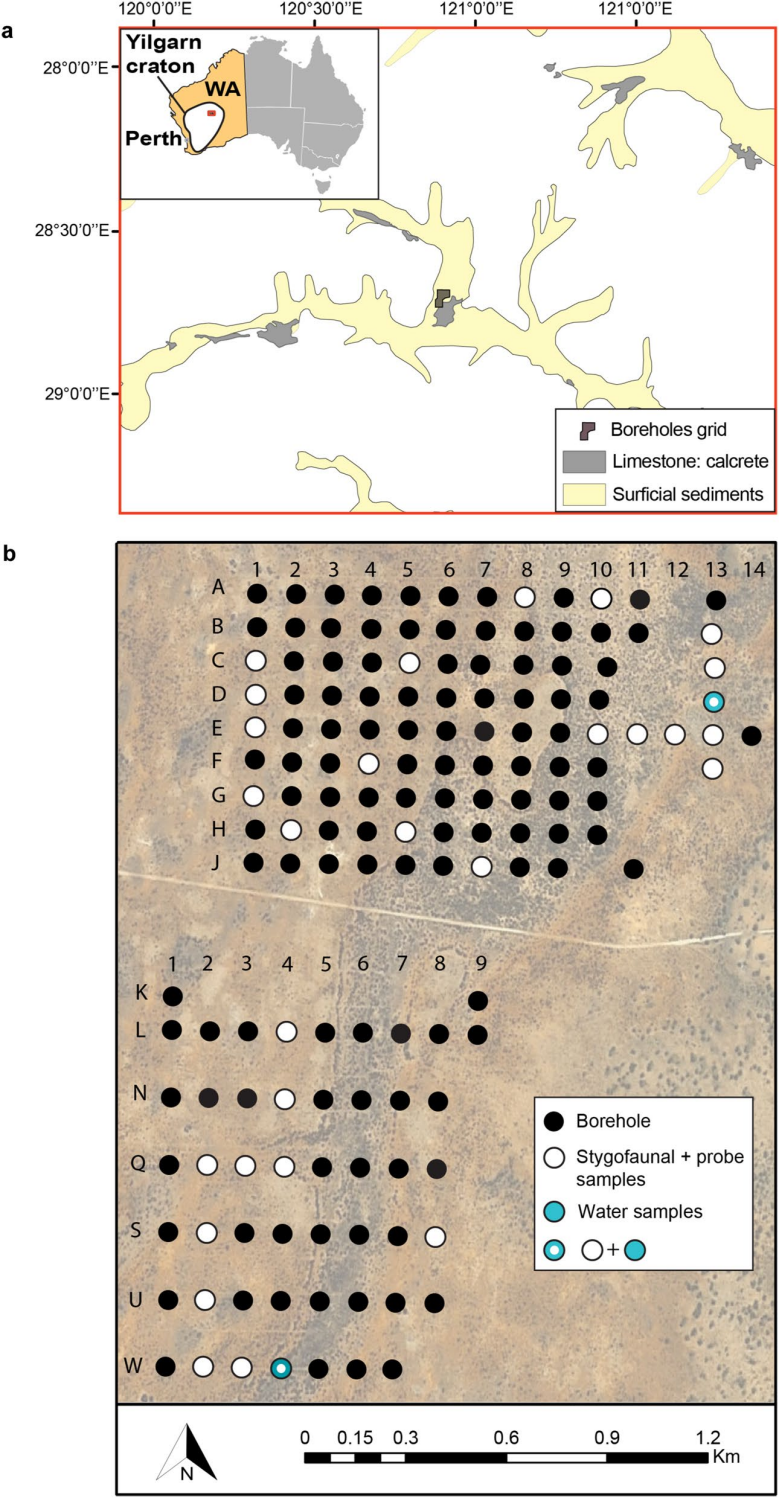
Map of the Sturt Meadows calcrete. (**a**) Location within the Yilgarn craton region and detailed paleodrainages/cacretes in the area and (**b**) the grid map showing the location of the boreholes sampled for stygofauna together with probe samples, water samples (in light blue) and the combination of both. Map was produced in ArcGIS Desktop 10.6^79^ and edited in Adobe Illustrator 25.0^80^.

The field work was carried out at the Sturt Meadows calcrete aquifer (28°41′ S 120°58′ E) located on Sturt Meadows pastoral station, Western Australia, ~ 42 km from the settlement of Leonora (833 km northeast of Perth, see Fig. 1a). The study area is a calcrete aquifer lying in the Raeside paleodrainages in the Yilgarn region of Western Australia (Fig. 1a). The vegetation of the area is Acacia woodlands, primarily *Acacia aneura* (F.Muell. ex Benth.), and is subjected to combined grazing pressure from domestic stock, feral animals and macropods. The aquifer is accessible through a bore grid comprising 115 bore holes of between 5 and 11 m depth (Fig. 1b).

Three sampling campaigns were carried out, two of them (LR1: 26/07/2017 and LR2: 07/11/2017) corresponding to low rainfall periods^24^ and one during the wet season (high rainfall, HR; two consecutive days of sampling collection: 17-18/03/2018) (Supplementary Fig. 1). The well-studied stygofaunal community of the area is composed of 11 main stygofaunal taxa belonging to five Classes: Oligochaeta (family Tubificidae (Vejdovský 1884)), subcohort Hydrachnidia, Maxillopoda (two species of harpacticoids: *Novanitocrella cf. aboriginesi* (Karanovic, 2004), *Schizopera cf. austindownsi* (Karanovic, 2004) and four species of cyclopoids: *Halicyclops kieferi* (Karanovic, 2004), *Halicyclops cf. ambiguous* (Kiefer, 1967), *Schizopera slenderfurca* (Karanovic & Cooper, 2012) and *Fierscyclops fiersi* (De Laurentiis et al., 2001)), Malacostraca: Amphipoda (species *Scutachiltonia axfordi* (King, 2012), *Yilgarniella sturtensis* (King, 2012) and *Stygochiltonia bradfordae* (King, 2012)) and Insecta: Coleoptera: Dytiscidae (species *Paroster macrosturtensis* (Watts & Humphreys 2006), *Paroster mesosturtensis* (Watts & Humphreys 2006) and *Paroster microsturtensis* (Watts & Humphreys 2006) and respective larvae).

### Field work procedures and sample preparation

Given the sensitivity of the hydrological dynamics in shallow calretes^23,25^, extensive water extraction along the bores was avoided, and preliminary tests on the bores with the highest water depth were carried out to quantify potential risk of dewatering the calcrete. During the field campaigns LR2 and HR, 20 water samples in total (two samples for stable isotope analysis on DOC (Dissolved Organic Carbon) and DIC (Dissolved Inorganic Carbon), three samples for radiocarbon analysis on DOC, one sample for radiocarbon analysis on DIC, and two samples for stable isotope and radiocarbon analyses on POC (Particulate Organic Carbon)) were collected from bores D13 and W4 (Fig. 1b), which are representative of the two main geological conformations of the area—calcrete (W4) and clay (D13) (Supplementary Fig. 2)—and host stable hydrological and biotic conditions^7^. Water samples were collected using a submersible centrifugal pump (GEOSub 12 V Purging Pump) after wells were purged of three well-volumes and stabilisation of in-field parameters was observed, according to the methodology in Bryan et al*.*^26^.

Samples for ^14^C_DIC_ analysis were filtered through 0.45 μm filters and collected in 1 L high density poly-ethylene (HDPE) bottles. δ^13^C_DIC_ samples were filtered through 0.2 μm filters, collected in 12 mL glass vials (Exetainers) and refrigerated after sampling. δ ^13^C_DOC_ samples were filtered through 0.2 μm filters, collected in 60 mL HDPE bottles and frozen after sampling.^14^C_DOC_ samples were filtered through 0.2 μm filters, collected in 3 L HDPE bottles and frozen after sampling.

In order to investigate ^14^C and δ ^13^C content of POC, two extra liters were collected from the same bores (D13, W4) and kept frozen (− 20 °C) until further analyses. ^14^C_POC_ δ^13^C_POC_ samples were then filtered through pre-combusted GF/F filters (12 h at 450 °C), washed with 1.2 N HCl to remove any inorganic carbon, and subsequently dried at 60 °C for 24 h. All samples were closed with sealing tape after collection to limit atmospheric exchange and kept in darkness.

Temperature, pH, ORP, salinity, DO and depth were measured in situ using a portable Hydrolab Quanta Multi‐Probe Meter across 30 bores during LR1, LR2 and HR^23^ (presented in Supplementary Table 8). Adult and larval stygofaunal specimens were collected from the same bores by hauling a weighted plankton net (mesh 100 μm^27^) five times through the water column (Fig. 1b). All biological samples were kept frozen (− 20 °C) in darkness until laboratory processing. Individual organisms were counted and identified (and consequently separated) to the lowest taxonomic level via optical microscopy and reference to specific taxonomic keys. Plant material, sediment samples and fauna were each separated during the sorting in the laboratory and each taxon pooled according to sampling campaign (LR1, LR2 or HR) and subsequently washed with Milli-Q water to remove surface impurities from their bodies. Sediment samples were soaked in acid (0.1 N HCl) to remove inorganic carbon, and together with the other samples were then oven dried at 60 °C overnight and ground until obtaining a homogeneous fine powder and stored at − 20 °C until further analyses.

Previous investigations on the ecological niche trends at Sturt Meadows indicated that all stygofauna characterize similar niche occupations under low rainfall regimes (LR1 and LR2)^23^. Stygofaunal specimens from the two low rainfall sampling events were combined to form sample LR to address the competing requirements between isotopic detection limits, analytical replicates and cost, while maintaining the main taxonomic and biological classifications^7^.

### Bulk isotope and ^14^C analyses

Water δ^13^C_DIC_ and δ^13^C_POC_ isotopic ratios were analysed by Isotope Ratio Mass Spectrometer—WABC at The University of Western Australia using a GasBench II coupled with a Delta XL Mass Spectrometer (Thermo-Fisher Scientific)—and the results, with a precision of ± 0.10‰, were reported as ‰ deviation from the NBS19 and NSB18 international carbonate standard^28^.

δ^13^C_DOC_ isotopic ratios of waters were analysed via Liquid Chromatography Isotope Ratio Mass Spectrometer (LC-IRMS) at the La Trobe Institute for Molecular Sciences (LIMS, La Trobe University, Melbourne, Australia) comprising an Accela 600 pump connected to a Delta V Plus Isotope Ratio Mass Spectrometer via a Thermo Scientific LC Isolink (Thermo Scientific).

C and N bulk SIA on homogenised samples of sediment, roots, stygofauna and copepods (cyclopoids and harpacticoids) were performed at the Australian Nuclear Science and Technology Organisation (ANSTO, Sydney, Australia). Samples were loaded into tin capsules and analysed with a continuous flow isotope ratio mass spectrometer (CF-IRMS), model Delta V Plus (Thermo Scientific Corporation, U.S.A.), interfaced with an elemental analyser (Thermo Fisher Flash 2000 HT EA, Thermo Electron Corporation, USA) following the procedure published by Mazumder et al*.*^29^.

For radiocarbon analyses, samples (sediment, roots, copepods, ants, stygofauna) were treated with 1 M HCl for 2 h to remove all possible carbonate contamination. These pre-treated samples together with ^14^C_POC_, ^14^C_DOC_ and ^14^C_DIC_ samples were subjected to CO_2_ extraction and graphitization following the methodology published by Hua et al*.*^30^. ^14^C content of samples was determined by means of the Accelerator Mass Spectrometry (AMS) at ANSTO.

### Carbon CSIA

Carbon CSIA followed the procedure described in Saccò et al*.*^7^. Samples of roots and stygofaunal specimens were hydrolysed under vacuum with 0.5 to 1 mL of amino acid-free 6 M HCl (Sigma-Aldrich) at 110 °C for 24 h. The protein hydrolysates were dried overnight in a rotary vacuum concentrator and stored in a freezer. Prior to analysis, the samples were dissolved in Milli-Q water and 10 μL of 1-mmol solution of 2-aminoisobutyric acid (Sigma-Aldrich) as internal standard. The sample stock had a concentration of approximately 8 to 10 mg/mL, which was further diluted as needed. Single amino acid carbon isotope analysis was carried out at the La Trobe Institute for Molecular Sciences (LIMS, La Trobe University, Melbourne, Australia) using an Accela 600 pump connected to a Delta V Plus Isotope Ratio Mass Spectrometer via a Thermo Scientific LC Isolink (Thermo Scientific).

The amino acids were separated using a mixed mode (reverse phase/ion exchange) Primesep A column (2.1 × 250 mm, 100 °C, 5 μm, SIELC Technologies) following the chromatographic method described in Mora et al*.*^31^ after Smith et al*.*^32^. Mobile phases are those described in Mora et al*.*^33^. Percentage of Phases B and C in the conditioning run, as well as flow rate of the analytical run and timing of onset of 100% Phase C were adjusted as needed. Samples were injected onto the column in the 15 μL—partial loop or no waste—injection mode, and measured in duplicate or triplicate.

To elucidate carbon flows through the stygofaunal community we focused on the essential amino acids Valine (Val), Phenylalanine (Phe) and Arginine (Arg), as these compounds must be integrated through diet and cannot be synthetised internally by the fauna^14,34^. In addition, to distinguish between terrestrial and aquatic carbon sources, the ratio between Val and Phe signals (δ^13^C_Val-Phe_), a widely employed index in archaeology and freshwater biology^35^, was calculated for roots, water mites, aquatic worms, amphipods and beetles (larvae and adults).

### Microbial taxonomic and functional gene analyses

Consumer amphipods (*Scutachiltonia axfordi* (AM1), *Yilgarniella sturtensis* (AM2), *S. bradfordae* (AM3)), cyclopoids and harpacticoids, together with predator stygobiotic beetles (*Paroster macrosturtensis* (B), *P. mesosturtensis* (M) and *P. microsturtensis* (S)) (see Saccò *el at* ^7^*.* for further details on the trophic characterisation of the stygofaunal community at Sturt Meadows), were used for gut microbiome bacterial 16S metabarcoding and microbial functional analysis. A total of 16 AM1, 16 AM2, 16 AM3, 20 cyclopoids and 20 harpaticoids and 20 of each one of the three *Paroster* species (B, M and S), were sorted into duplicates of stygobiotic pools of 3–5 individuals from both LR and HR events for DNA extraction. Prior to DNA extraction stygobitic animals (3–5 individuals per pool; n = 40) were placed in a petri dish containing ultrapure water and UV sterilized in a UV oven for 10 min to eliminate any bacterial species that may be contained on the exoskeleton as this study targeted the gut microbiome. Immediately post-UV treatment, the animals were placed into tissue lysis tubes with 180 μL tissue lysis buffer (ATL) and 20 μL Proteinase K and homogenised using Minilys tissue homogeniser (ThermoFisher Scientific, Australia) at high speed for 30 s. Lysis tubes, inclusive of two laboratory controls, were incubated at 56 °C using an agitating heat block (Eppendorf ThermoStat C, VWR, Australia) for 5 h.

Following the incubation, the analytical procedure was adapted from Saccò et al*.*^22^ and DNA extraction was carried out using DNeasy Blood and Tissue Kit (Qiagen; Venlo, Netherlands) and eluted off the silica column in 30–50 μL AE buffer. The quality and quantity of DNA extracted from each stygobitic pool was measured using quantitative PCR (qPCR), targeting the bacterial 16S gene. PCR reactions were used to assess the quality and quantity of the DNA target of interest via qPCR (Applied Biosystems [ABI], USA) in 25 μL reaction volumes consisting of 2 mM MgCl_2_ (Fisher Biotec, Australia), 1 × PCR Gold Buffer (Fisher Biotec, Australia), 0.4 μM dNTPs (Astral Scientific, Australia), 0.1 mg bovine serum albumin (Fisher Biotec, Australia), 0.4 μM of each primer (Bact16S_515F and Bact16S_806R^36,37^), and 0.2 μL of AmpliTaq Gold (AmpliTaq Gold, ABI, USA), and 2 μL of template DNA (Neat, 1/10, 1/100 dilutions). The cycling conditions were: initial denaturation at 95 °C for 5 min, followed by 40 cycles of 95 °C for 30 s, 52 °C for 30 s, 72 °C for 30 s, and a final extension at 72 °C for 10 min.

DNA extracts that successfully yielded DNA of sufficient quality, free of inhibition, as determined by the initial qPCR screen (detailed above), were assigned a unique 6–8 bp multiplex identifier tag (MID-tag) with the bacterial 16S primer set. Independent MID-tag qPCR for each stygobiotic pool were carried out in 25 μL reactions containing 1 × PCR Gold Buffer, 2.5 mM MgCl_2_, 0.4 mg/mL BSA, 0.25 mM of each dNTP, 0.4 μM of each primer, 0.2 μL AmpliTaq Gold and 2–4 μL of DNA as determined by the initial qPCR screen. The cycling conditions for qPCR using the MID-tag primer sets were as described above. MID-tag PCR amplicons were generated in duplicate and the library was pooled in equimolar ratio post-PCR for DNA sequencing. The final library was size selected (160–600 bp) using Pippin Prep (Sage Sciences, USA) to remove any MID-tag primer-dimer products that may have formed during amplification. The final library concentration was determined using a QuBitTM 4 Fluorometer (Thermofischer, Australia) and sequenced using a 300 cycle V2 kit on an Illumina MiSeq platform (Illumina, USA).

MID-tag bacterial 16S sequence reads obtained from the MiSeq were sorted (filtered) back to the stygobitic pool based on the MID-tags assigned to each DNA extract using Geneious v10.2.5^38^. MID-tag and primer sequences were trimmed from the sequence reads allowing for no mismatch in length or base composition.

Filtered reads were then input into a containerised workflow comprising USEARCH^39^ and BLASTN^40^, which was run on a high-throughput HPC cluster at Pawsey supercomputing centre. The fastx-uniques, unoise3 (with minimum abundance of 8) and otutab commands of USEARCH were applied to generate unique sequences, ZOTUs (zero-radius Operational Taxonomic Units) and abundance table, respectively. The ZOTUs were compared against the nucleotide database using the following parameters in BLASTN: perc_identity ≥ 94, evalue ≤ 1e−3, best_hit_score_edge 0.05, best_hit_overhang 0.25, qcov_hsp_perc 100, max_target_seqs = 5. An in-house Python script was used to assign the ZOTUs to their lowest common ancestor (LCA)^41^. The threshold for dropping a taxonomic assignment to LCA was set to perc_identity ≥ 96 and the difference between the % of identity of the two hits when their query coverage is equal was set to 1. Results on the microbial taxonomic diversity detected in ground water samples from a previous study on carbon inputs in the aquifer^22^ were incorporated in this work to allow qualitative comparison with the stygofaunal gut diversity.

To investigate functional activity involved in carbon cycling, the 16S metabarcoding data were fed to the Phylogenetic Investigation of Communities by Reconstruction of Unobserved States 2 (PICRUSt2) software package to generate predicted metagenome profiles^42^. These profiles were clustered into Kyoto Encyclopedia of Genes and Genomes (KEGG) Orthologs (KOs)^43^ and MetaCyc pathway abundances^44^ focusing on the relative abundances of four carbon metabolisms: carbon fixation in prokaryotes, carbohydrates, lipids and amino acid metabolisms. Relative abundance of pathways linked with methane, nitrogen and sulfur metabolisms were also investigated.

### Statistical analyses

The Phyloseq package in R^45,46^ was used to plot the ZOTU abundance for the styfofaunal specimens at the order level under LR and HR periods. The Statistical Analysis of Metagenomic Profiles (STAMP) bioinformatics software package was used to carry out Principal Components Analysis (PCA) to assess the differences between functional genomic fingerprints based on the KEGG orthologs function prediction between copepods (C and H) and amphipods (AM1, AM2 and AM3), and determine statistically significant results from the PICRUSt2 output^47^. Clustering patterns according to rainfall periods (LR and HR) and major consumers taxonomic groups (cyclopoids, harpacticoids and amphipods) were assessed through Permutational multivariate analysis of variance (PERMANOVA, R-package^46^ ‘vegan’) and pairwise post hoc pairwise multilevel comparisons^48^.

For comparison of potential shifts in abundances of microbial metabolic pathways within groundwater samples, copepods and amphipods across rainfall periods, analysis of variance (ANOVA) was performed on the abundance data (two replicates per each group) on the predicted pathways depicting carbon fixation, carbohydrate, lipid, amino acid, methane, nitrogen and sulfur metabolisms. ANOVAs coupled with Tukey’s HSD pairwise comparisons (R-package^46^ ‘stats’) were employed to inspect significant differences between bulk SIA (δ^13^C and δ^15^N) and essential amino acid (δ^13^C_Phe_, δ^13^C_Arg_, δ^13^C_Val_ and δ^13^C_Val-Phe_) data from the stygofaunal taxa within the different rainfall conditions (LR and HR). PERMANOVAs (R-package^46^ ‘vegan’) were also performed to investigate the potential clustering trends within the stygofaunal taxa across rainfall periods from the combination of radiocarbon (Δ^14^C) and carbon SIA (δ^13^C) isotopic fingerprints.

SIMM (Stable Isotope Mixing Models, R-package^46^ ‘simmr’) were used to estimate dietary proportions of copepods and amphipods within a Bayesian framework. Due to the lack of species-specific trophic discrimination factors for stygofauna, we employed the widely accepted values of 3.4 ± 2‰ for nitrogen and 0.5 ± 1‰ for carbon^49^. Markov chain Monte Carlo (MCMC) algorithms were used for simulating posterior distributions in SIMM, and MCMC convergence was evaluated using the Gelman-Rubin diagnostic by using 1.1 as a threshold value for analysis validation.

## Results

### Stygofaunal gut microbiome patterns

**Figure 2:**
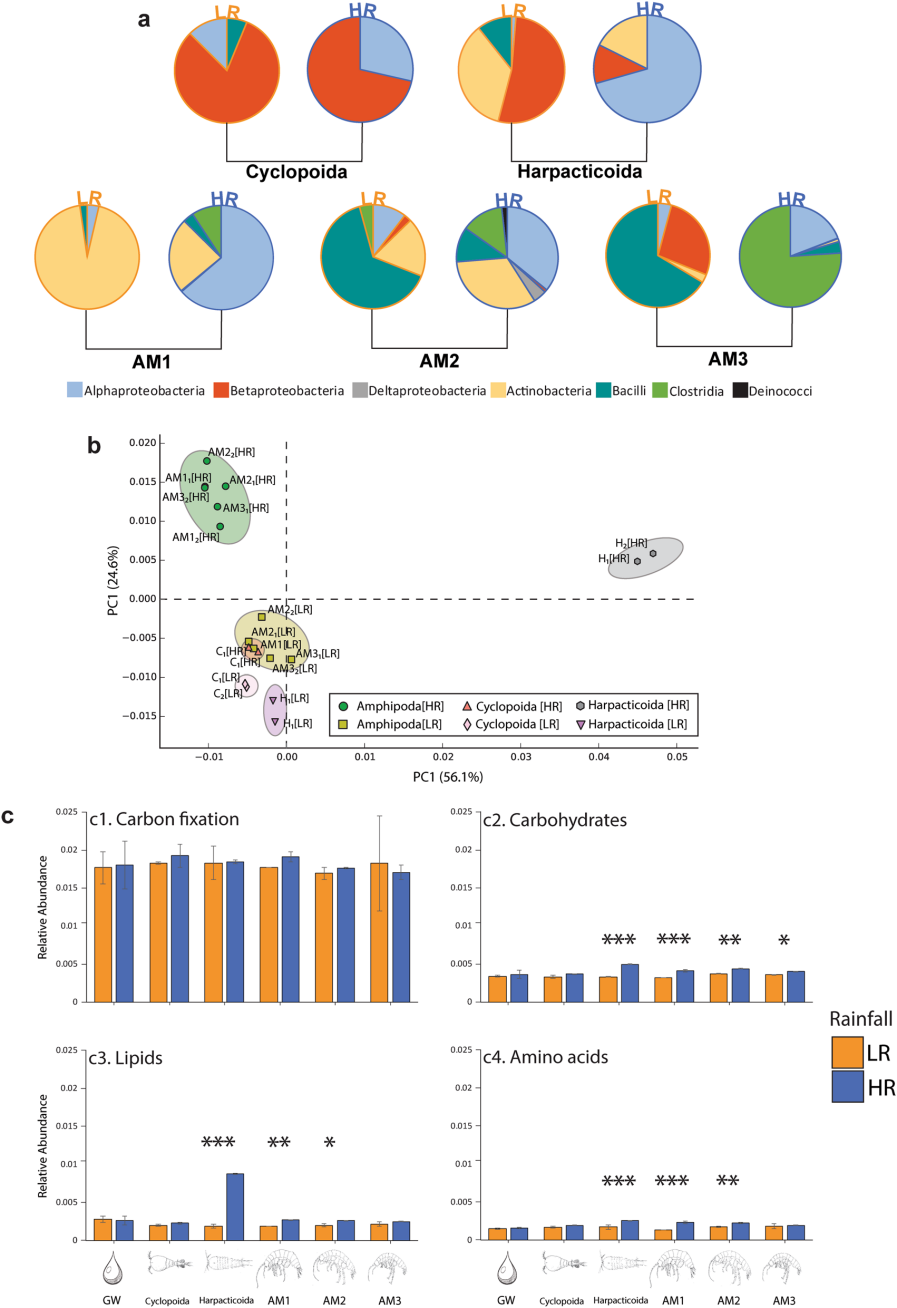
Microbial gut diversity and functional genomic results. (**a**) Relative abundances (in %) of the classes found in copepods (cyclopoids and harpacticoids) and amphipods AM1, AM2 and AM3 under LR (low rainfall) and HR (high rainfall). (**b**) PCA-based ordination analysis illustrating the distribution of taxa across rainfall periods (LR and HR) according to the KEGG orthologs metabolic functions. (**c**) Abundances of the major KEGG pathways associated with carbon metabolism (c1), carbohydrates metabolism (c2), lipids metabolism (c3) and amino acids metabolism (c4). *P < 0.05; **P < 0.005; ***P < 0.0005.

The gut microbiome of cyclopoids was dominated by betaproteobacteria under both rainfall regimes (accounting for 81% under LR and 71% under HR), while the microbiome community of harpacticoids illustrated a shift towards alphaproteobacteria (reaching 70% of the total) under HR. During LR, gut microbiomes of amphipods were dominated by the classes Actinobacteria (94% in AM1) and Bacilli (reaching 83% together with Actinobacteria in AM2 and 93% together with Betaproteobacteria in AM3). Conversely, the most abundant classes within amphipods under HR were Alphaproteobacteria (64% in AM1 and 36% in AM2) and Clostridia (ranging up to 95% together with Alphaproteobacteria in AM3) (Fig. 2a).

The PCA using the KEGG orthologs function prediction showed that cyclopoids from both rainfall periods (C[LR] and C[HR]) clustered close to the harpacticoids (H[LR]) and amphipods (AM1[LR], AM2[LR] and AM3[LR]) from the LR regime (Fig. 2b). In contrast, the latter two taxa grouped separately to the rest of the primary and secondary consumers under HR. Overall, the community clustered differently during the two rainfall periods (PERMANOVA, *P* < 0.05) and also according to the separation in major consumers taxonomic groups (cyclopoids, harpacticoids and amphipods) across LR and HR (PERMANOVA, *P* < 0.005). However, pairwise comparisons showed no significant change across taxa and between the two rainfall events.

Predictions on the quantitative proportion of individual carbon metabolic pathways showed that carbon fixation was the most abundant metabolism within the four main routes analysed, accounting on average for 1.8% of the total, followed by carbohydrate (0.4%), lipid (0.3%) and amino acid (0.2%) metabolisms. Apart from AM3 (Fig. 2c.1), all the taxa illustrated increasing trends in abundance of the cited carbon metabolisms activity after rainfall (HR). Carbohydrate, lipid and amino acids metabolic categories significantly increased in harpacticoids, AM1 and AM2 during HR (Figs. 2c.2, 3 and 4), whilst only abundances of predicted pathways associated with carbohydrate metabolism increased in AM3 (Fig. 2c.2).

**Figure 3:**
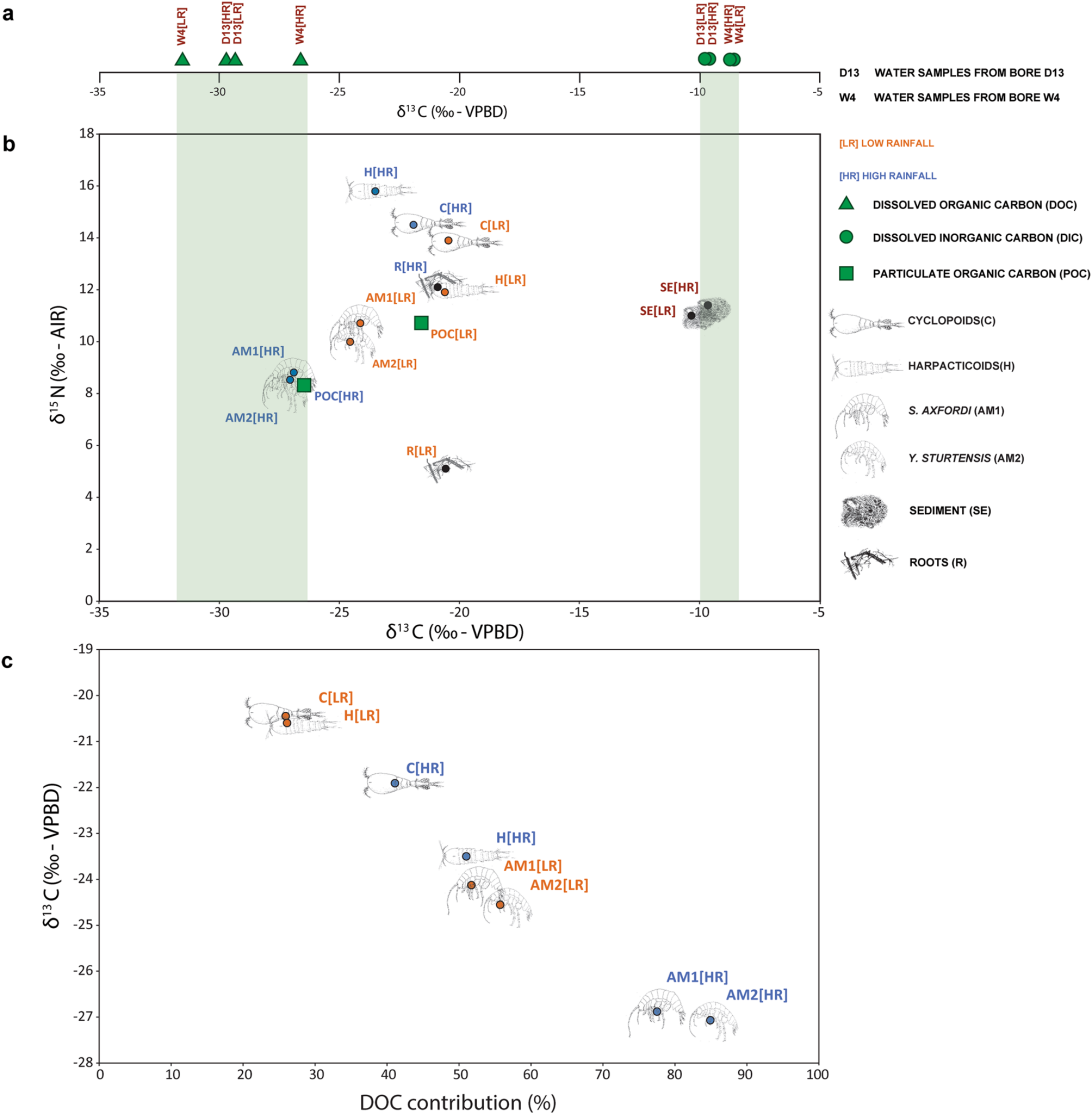
Isotopic patterns amongst primary and secondary consumers and their carbon sources. (**a**) δ^13^C_DOC_ and δ^13^C_DIC_ from the bores W4 and D13 (during LR and HR) and their ranges (in light green) incorporated in graph (**b**), which illustrates the δ^13^C and δ^15^N for LR (low rainfall) and HR (high rainfall) of roots, sediment, POC (particulate organic matter), copepods (C and H) and amphipods (AM1 and AM2); VPBD: Vienna Pee Dee Belemnite; AIR: N_2_ of atmospheric air; in red the old (considering present as 1950) carbon sources revealed by radiocarbon dating (refer to Table 1 for specific values). (**c**) Estimation of DOC contributions for the diets of copepods (C and H) and amphipods (AM1 and AM2) during LR and HR.

**Figure 4:**
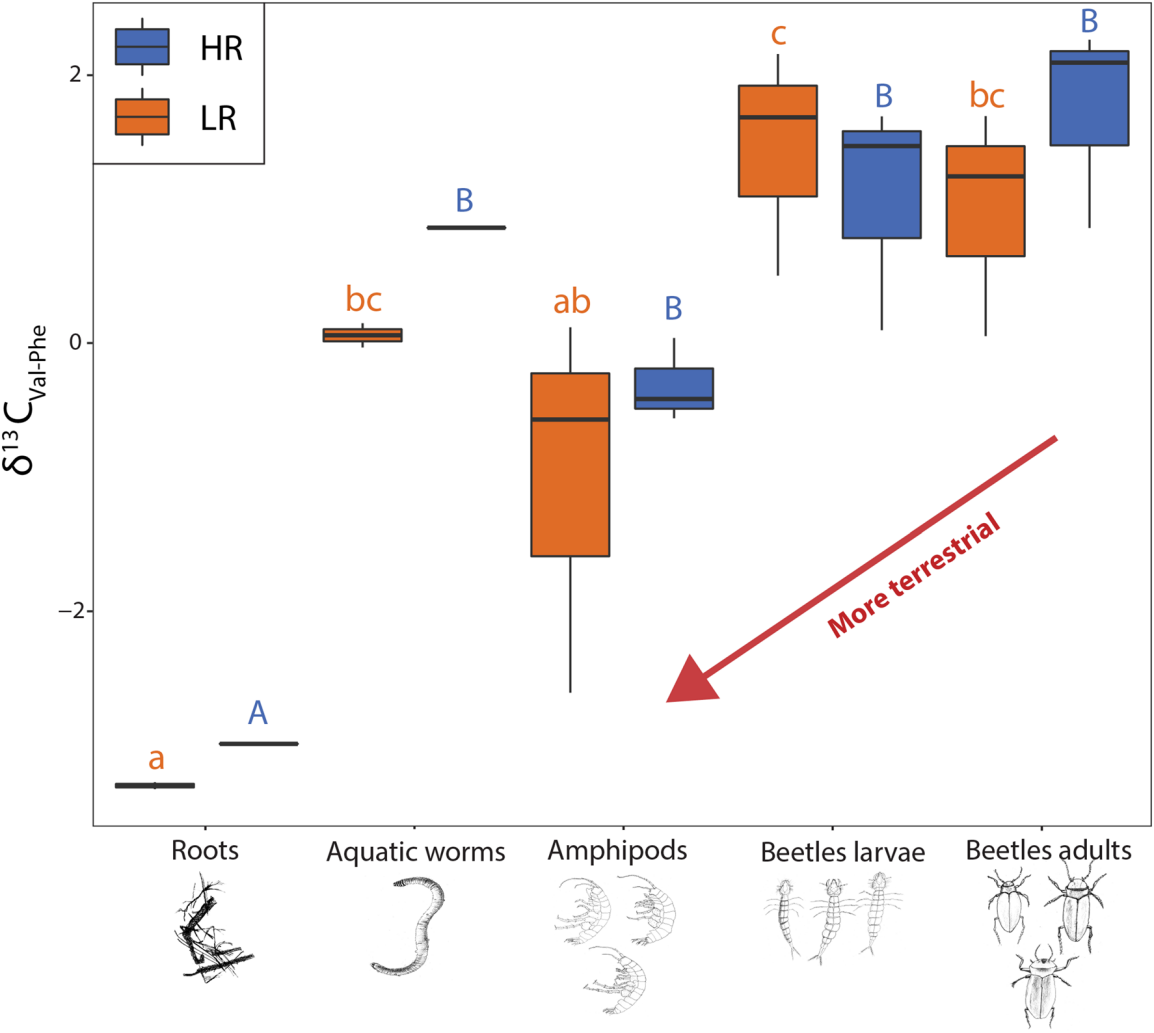
δ^13^C_Phe-Val_ values calculated under LR and HR conditions for roots, aquatic worms, amphipods (AM1, AM2 and AM3), beetles larvae (Blv, Mlv, Slv) and adults (B, M, S). More negative values indicate a more terrestrial carbon source. Refer to Supplementary Table 2 and Supplementary Table 3 for the significances of the pairwise comparisons. Letters a,b and c are used to indicate significantly different δ^13^C_Phe-Val_ values (*P* < 0.05) for LR conditions, while letters A, B and C are for HR for the same purpose.

### Biochemical flows and stygofauna

#### Organic inputs

The bulk isotopic value of δ^13^C and δ^15^N of sediment (the organic fraction from LR and HR) were similar to each other (Table 1) and both revealed very similar δ^13^C values to the DIC (Fig. 3b). Both groundwater sediment and DIC depicted old carbon sources within the two rainfall periods (Fig. 3a).

**Table 1:** Results from the δ^13^C, δ^15^N and ^14^C analyses on *Scutachiltonia axfordi* (AM1), *Yilgarniella sturtensis* (AM2) copepods (cyclopoids and harpacticoids), sediment, roots, particulate organic carbon (POC), dissolved inorganic carbon (DIC) and dissolved organic carbon (DOC).

	δ^13^C		δ^15^N		LR			HR		
	LR	HR	LR	HR	pMC	Δ^14^C (‰)	Age (BP)	pMC	Δ^14^C (‰)	Age (BP)
*S. axfordi*	− 24.14	− 26.88 ± 0.05	10.71	8.81 ± 0.09	102.84 ± 0.48	19.9 ± 4.8	Modern	99.86 ± 1.01	− 9.6 ± 9.9	Modern
*Y. sturtensis*	− 24.55	− 27.10	9.99	8.50	100.46 ± 0.36	− 3.7 ± 3.6	Modern	100.75 ± 0.81	− 0.9 ± 8.1	Modern
Cycopoida	− 20.45 ± 0.30^a^	− 21.91 ± 0.30^a^	13.90 ± 0.30	14.5 ± 0.30	100.27 ± 0.56^c^	− 5.6 ± 5.6^c^	Modern^c^	99.36 ± 0.68^c^	− 14.7 ± 6.7^c^	Modern^c^
Harpacticoida	− 20.60 ± 0.30^a^	− 23.50 ± 0.30^a^	11.90 ± 0.80	15.8 ± 0.80						
Roots	− 20.57 ± 0.30^a^	− 20.90 ± 0.30^a^	5.10 ± 2.0	12.1 ± 0.30	103.63 ± 0.30	27.7 ± 3.0	Modern	103.34 ± 0.47	24.9 ± 4.7	Modern
Sediment	− 10.33 ± 0.30^a^	− 9.65 ± 0.30^a^	11.0 ± 1.20	11.4 ± 1.20	22.16 ± 3.23	− 780.3 ± 32.1	12,100 ± 1170	57.68 ± 5.21	− 428.0 ± 51.7	4420 ± 725
POC	− 21.58 ± 0.10^b^	− 26.47 ± 0.10^b^	10.73 ± 0.10^b^	8.35 ± 0.10^b^	86.55 ± 0.76	− 141.7 ± 7.6	1160 ± 70	63.86 ± 0.28	− 366.7 ± 2.8	3605 ± 35
DOC_D13_	− 29.25 ± 0.36	− 29.35 ± 0.20	na	na	91.70 ± 0.46	− 90.6 ± 4.6	695 ± 45	92.41 ± 0.62	− 83.5 ± 6.1	630 ± 60
DOC_W4_	− 31.91 ± 0.50	− 27.15 ± 0.03	na	na	66.76 ± 0.48	− 337.9 ± 4.8	3245 ± 60	57.99 ± 0.55	− 424.9 ± 5.5	4380 ± 80
DIC_D13_	− 9.45 ± 0.10^b^	− 9.39 ± 0.10^b^	na	na	82.73 ± 0.30	− 179.5 ± 3.0	1575 ± 30	77.90 ± 0.19	− 227.4 ± 1.9	2005 ± 20
DIC_W4_	− 8.75 ± 0.10^b^	− 8.87 ± 0.10^b^	na	na	62.47 ± 0.21	− 380.5 ± 2.1	3835 ± 30	59.20 ± 0.17	− 412.8 ± 1.7	4210 ± 25

Mean values ± standard deviations are illustrated; pMC: percent of modern carbon, with values higher than 100% generated as a result of the the Bomb Peak calibration process; BP: before present (with present as 1950).

^a^Accuracy of the CF-iRMS.

^b^Accuracy of the GC-iRMS.

^c^Calculated as overall copepods (cyclopoids mixed with harpacticoids).

Compared to sediment, roots had more depleted δ^13^C values (LR: δ^13^C =  −20.6‰; HR: δ^13^C =  − 20.9‰) and modern ^14^C fingerprints (Table 1), suggesting a recent terrestrial origin. In addition, a shift in δ^15^N content can be observed in roots between LR and HR conditions, varying from 5.1 ± 2.0‰ under LR to 12.1 ± 0.3‰ after rainfall (HR). During HR, POC had more depleted δ^13^C values (Fig. 3a) than for the LR period, together with consistently older ages (Table 1).

Copepods (Cyclopoida (C) and Harpacticoida (H)) illustrated close δ^13^C fingerprints to roots (cyclopoids: δ^13^C =  −20.5‰ during LR, δ^13^C =  −21.9‰ under HR; harpacticoids: δ^13^C =  −20.6‰ during LR, δ^13^C =  −23.5‰ under HR), while amphipods *S. axfordi* (AM1) and *Y. sturtensis* (AM2) showed more depleted values overall (Table 1). Moreover, copepods (C and H in one unique pool) and AM1 showed more depleted Δ^14^C values under HR conditions than during the dry season (LR).

Within copepods, the highest proportion of carbon assimilation under LR was derived from attached bacteria (32.3% for cyclopoids and 31.9% for harpacticoids), while during the same rainfall regime DOC was the major organic driver (~ 50%) within amphipods *S. axfordi* (AM1) and *Y. sturtensis* (AM2). Under HR conditions, microbially-derived DOC was incorporated at considerably higher proportions for both groups (41.1% and 51% for copepods (C and H), and 77.5% and 84.9% for amphipods (AM1 and AM2)) (Fig. 3c). These results suggest that during HR the groundwater ecosystem gets an inflow of rainfall that triggers ‘pulses’ of carbon and nutrients ultimately consumed by copepods (C and H) and amphipods (AM1 and AM2).

#### Carbon transfers

δ^13^C_Phe_, δ^13^C_Arg_ and δ^13^C_Val_ values indicated that most of the taxonomic groups showed significant seasonal change in their organic fingerprint (Supplementary Table 1). *P. microsturtensis* (S) and *S. bradfordae* (AM3) were the only taxa that did not change significantly between the rainfall periods (LR vs HR) for all three essential amino acids (Val, Arg and Phe), with this trend potentially attributable to coupled feeding habits (prey-predator interactions) or a highly conservative tendency in carbon assimilations for both groups.

The pattern unveiled by the analysis of δ^13^C_Val-Phe_ values under LR and HR conditions confirms the shift in carbon source path (Supplementary Table 2and 3; Fig. 4). During the dry season (LR), amphipods’ (pool of AM1, AM2 and AM3) carbon sources were not significantly different to root signatures. In contrast, beetle larvae and adults had significantly different δ^13^C_Val-Phe_ values, suggesting a more aquatic (stygofaunal based) preference in carbon incorporation, as would be expected in predators. Under the high rainfall regime (HR), δ^13^C_Val-Phe_ values for roots were significantly different (Fig. 4) from all the five stygofaunal groups.

The combination of radiocarbon (Δ^14^C) and carbon SIA (δ^13^C) fingerprints indicated that roots, copepods, amphipods and beetles grouped differently (PERMANOVA, *P* < 0.05), suggesting trophic niche partitioning processes in OM assimilation. Roots clustered separately to the rest of the samples (Supplementary Fig. 3) and together with adult beetles (B, M and S) and AM2 were the only taxa that showed comparable δ^13^C fingerprints across the rainfall regimes (LR and HR) (Table 1 and Supplementary Table 4). Conversely, amphipod AM1 and copepods (cyclopoids pooled together with harpacticoids) illustrated the biggest shifts in organic input preferences towards more depleted Δ^14^C and δ^13^C values (Supplementary Fig. 3; Table 1).

## Discussion

### Microbial/stygofauna transitions

Rainfall events are responsible for both carbon and nutrient infiltrations that play a key role in shaping biochemical dynamics in the Sturt Meadows calcrete aquifer^7,23^. Metabarcoding and predicted metagenome results show that the gut microbiomes of primary consumers—copepods, harpacticoids and amphipods—changed dramatically both in community composition and metabolic functions under HR. The significant increase in oligotrophic bacteria (Alphaproteobacteria and Clostridia) during HR suggests that these two bacterial phyla become more prevalent when greater amounts of dissolved organic matter and nutrients become available. This is consistent with previous studies that show these bacterial phyla to be the most common organic compound degraders found in aquifers^50–52^. These phyla dominated the gut microbiota of the harpacticoids and all the three amphipod species AM1, AM2 and AM3 during HR.

Overall, amphipods hosted more abundant microbial communities (322 individuals on average between AM1, AM2, AM3 during LR, and 1182 under HR) when compared to the bacteria found in water (Supplementary Fig. 4 and Supplementary Table 5). This is not surprising, in light of the dilution effect that water provides to the free-living bacteria^53^, and because stygofauna act as vectors for prokaryotes^54^.

Evidence from functional genomic analyses indicates that while carbon fixation pathways represent a stable metabolic baseline under both rainfall conditions, the abundance of carbohydrate, lipid and amino acid metabolisms significantly increased under HR within consumers such as harpacticoids and amphipods. Interestingly, within the primary consumer copepods, although harpacticoids showed consistent increased abundances of all the metabolisms studied after rainfall, those of cyclopoids remained steady. Galassi et al*.*^55^ reported that within low-water velocity karst systems, cyclopoids usually have free-swimming nektonic lifestyles, while harpacticoids prefer interstitial voids in the sediment^56^. At Sturt Meadows, different ethological dynamics after rainfall would allow competency to be diminished in an environment with limited resource availability. In contrast to cyclopoids, the microbial gut microbiome community of harpacticoids experienced a shift towards more abundant alphaproteobacteria during HR as well as increased methane, nitrogen and sulfur microbiome metabolisms (Supplementary Fig. 5), suggesting that their feeding sources are markedly different from cyclopoids. However, our data from Bayesian mixing models showed little difference between the diets of the two groups (Supplementary Table 6), and mesocosm experiments will be necessary to confirm niche partitioning patterns.

**Figure 5:**
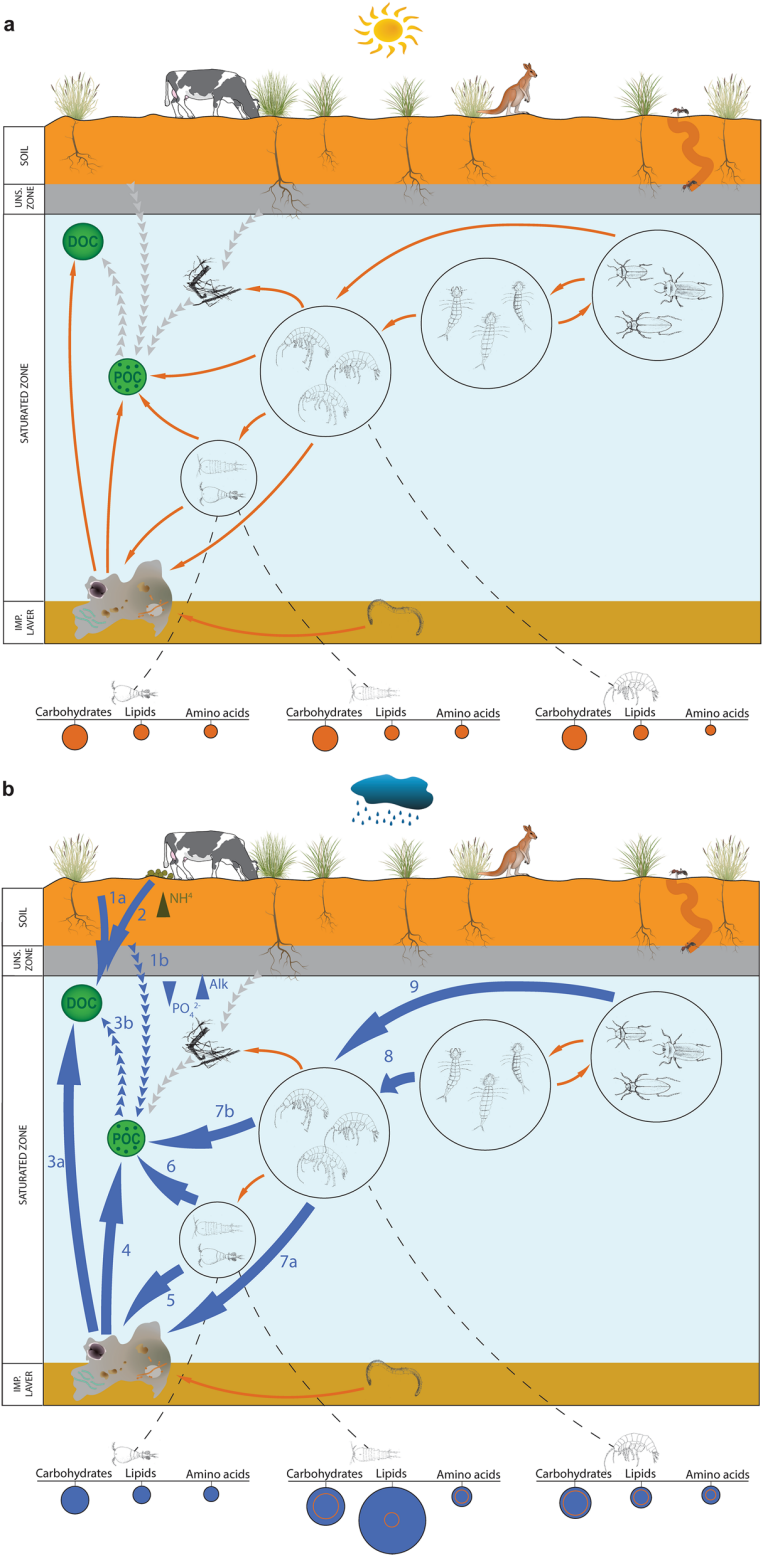
Conceptual model of the principal biochemical flows at Sturt Meadows aquifer under LR (**a**) and HR (**b**) conditions. The orange arrows illustrate the main biochemical paths, while bigger blue arrows underline those transitions that are strengthened under high rainfall period, and are numbered as follow: (1a) old and ^13^C-replenished DOC leaches into the groundwater (Table 1); (1b) as a result of the rainfall infiltration, phosphates dilute, carbonates are released (higher alkalinity)^23^ and old POC gets to the water; (2) ammonia concentrations increase as a combined effect of animal waste leaking from the surface and microbial metabolisms^22,23^); (3) microbial biofilms consume the newly incorporated old DOC (partially derived from POC (route 3b))^22^; (4) biofilms decompose POC; (5) harpacticoids browse on biofilm and cyclopoids filter particulate organic matter (route 6); (7a) amphipods graze on microbial mats (and filter POC (route 7b)) and fuel the carbon to the upper trophic levels; 8, beetles larvae and adults (route 9) (top predators) exert a higher trophic pressure on amphipods after rainfall^7^. Dashed lines lead to the proportions of the carbohydrate, lipid and amino acid microbial metabolisms (diameter of the bubbles proportional to the relative abundances in cyclopoids, harpacticoids and AM1; inner orange circles under HR (**b**) are illustrated for comparison with the significative lower relative abundances under LR (**a**)). Figure was produced and edited in Adobe Illustrator 25.0^80^.

Compared to copepods and amphipods, subterranean dytiscid species *P. mesosturtensis* (M) and *P. microsturtensis* (S) showed more uniform microbial gut communities (Supplementary Fig. 4) and more stable isotopic trends (Supplementary Fig. 3) across rainfall periods, indicating mitigated trophic shifts typical of constant predatory behaviors. Conversely, microbial gut diversity of *P. macrosturtensis* experienced a substantial shift from a Bacilli dominated environment under LR to an Actinobacteria-based community during HR, which might be ascribable to species-specific predatory pressures on AM1 and AM2 under HR^7^.

Overall, our results from genetic analyses on stygofaunal gut microbiomes suggest that the inflow of OM at Sturt Meadows is exploited by microbes which are the potential direct (through biofilm grazing) and indirect (via POC assimilation) diet sources of primary consumers, amphipods and copepods. A previous investigation on carbon inputs in water indicated a shift in microbial taxonomic assemblages coupled with increased degradative pathways after rainfall (HR)^22^. In line with our work, Reiss et al*.*^9^ reported that rainfall inflows, coupled with increased inputs of organic matter mediate changes in microbial diversity, abundances and respiration rates.

Meiofauna (copepods) and amphipods are commonly considered as filter-feeders and biofilm grazers^57,58^, and have been depicted as crucial actors in the carbon fueling to upper trophic levels^59^. In a recent study, Weitowitz et al*.*^60^ brought new light to the microbes-amphipods linkage, one of the most important associations in groundwater ecosystems, by providing empirical evidence of direct microbial ingestion by amphipods *Niphargus fontanus *(Bate, 1859) and *Niphargus kochianus *(Bate, 1859) and their resulting effects on biofilm assemblages.

Our study widens the understanding of these dynamics by incorporating novel information about the rainfall-driven shifts in functional metabolic activities of stygofaunal gut microbiomes. However, our molecular results, while interesting, are still indirect evidence of the ‘DOC-microbes-stygofauna’ ecological cascade, and community mesocosm experiments are critically needed. Indeed, further species-specific investigations are required to elucidate the mechanisms of these interactions and bring crucial comprehension of the dynamics sustaining groundwater biodiversity.

### Faunal trends: carbon paths and food web interactions

Australian shrubs potentially constitute a driver between surface and subsurface biochemical frameworks, especially in arid soils^61,62^. Mulga roots have been reported penetrating deep into the soil to reach moisture, and frequently fall from the unsaturated zone into aquifers^63^. At Sturt Meadows, barcoding analyses revealed that root fragments in the water match with saltbush vegetation from the surface (J. Hyde’s personal communication). Interestingly, while roots δ^13^C values did not change between rainfall regimes, δ^15^N showed more depleted values under LR (Table 1). Termites, widely distributed in the area, are wood feeders that could play a key role in nitrogen fixation^64^. Our results align to this hypothesis, with termites benefiting from the easily accessible nitrogen source from the nitrophilous mulga vegetation. In fact, we observed increased rates of nitrogen-depleted root material falling into the aquifer under environmental conditions (dry season, LR) which have been reported as favourable for termites’ ethology^65,66^. Concurrently, moisturised vegetal material is highly likely to be targeted by fungi and microbes^67^ in the hyporeic zone, and enriched δ^15^N values under HR might be a reflection of coexisting microbiological metabolisms^7^.

δ^13^C bulk values for roots, close to C3 photosynthetically-derived carbon fingerprints^68^, were almost identical to those of the meiofaunal and stygofaunal communities (Table 1). These results reflect the lack of potential trajectories of trophic increments (from roots to the top predators) found for the same system by Bradford et al*.*^20^, and suggest other paths of carbon assimilation. However, the inorganic carbon component (DIC) in water is only a marginal contributor to biological incorporation as it is probably sourced from calcrete bedrocks as indicated by very similar isotopic fingerprints to the sediment in both stable and radiocarbon data (sediment: Δ^14^C of − 428.0 ± 51.7‰ (HR) and δ^13^C values ranging from − 10.33‰ (LR) to − 9.65‰ (HR), see Table 1).

Several groundwater studies report terrestrially derived DOC as a primary factor in shaping ecological shifts under differential recharge conditions^6,9^. The δ^13^C DOC values detected in this study (ranging from − 31.91 ± 0.5‰ to − 27.15 ± 0.03‰) were characteristic of surface derived carbon sources (− 20‰ depleted if compared with atmospheric CO_2_ values of − 8‰^69^), suggesting that allochthonous material potentially drives the biochemical flows in the system. Interestingly, the Sturt Meadows stygofaunal community illustrated differential OM incorporations under LR and HR regimes. During the dry period (LR), isotopic evidence from amphipods revealed that microbially-derived DOC incorporations were combined with sediment (~ 20% contribution), POC (~ 20% contribution) and their attached microbial communities (Supplementary Table 6). This tendency towards opportunistic strategies shifts under HR, when biochemically enriched aquifers via rainfall inflows triggered a dominance of DOC-derived assimilations (ranging from 77.5% (AM1) to 84.9% (AM2)). Compared to amphipods, meiofauna (cyclopoids and harpacticoids) showed increased sediment ingestion under LR (~ 32% contribution), however the consistent increase in DOC incorporations was confirmed after rainfall (Supplementary Table 6). The δ^15^N signatures of stygofauna were consistent with a food web driven by soil-based OM incorporations^70^ and meiofauna illustrated anomalously enriched δ^15^N fingerprints compared to amphipods under both rainfall regimes. Moreover, cyclopoids and harpacticoids were the only groups which experienced increased δ^15^N values coupled with rainfall under the HR regime, suggesting different nitrogen microbial baselines^7,71^ coupled with potential scavenging^72^.

Stygobionts illustrate high resiliency rates to a lack of resources^73^. As reported by Gibert and Deharveng^74^, evolutionary trends in groundwater biota might have driven maximization of trophic plasticity coupled with low metabolic rates. Our results indicate that while under LR regimes omnivory might play a key role in maintaining stygofaunal assemblages, under HR conditions, prey-predator interactions—ultimately driven by shifts in trophic habits carried out by specific groups such as amphipods—are strengthened^7^. Amphipods display a vast array of feeding modes—from facultative biofilm grazers to scavengers—which is thought to be linked to their resistance to starvation^59,75^. From an eco-biochemical perspective, amphipods—when present in groundwater—are major components in microbial assimilation processes that fuel carbon transfers along the trophic chain^10^. We did not find direct isotopic evidence of biofilm assimilation from epilithic microbial biofilms, however we found that amphipods did shift towards ^13^C-depleted carbon sources, and potentially more microbially-derived OM, under HR condition (Fig. 3 and Supplementary Fig. 3). Aquatic worms (OL) showed a shift towards more depleted OM incorporations, however further isotopic and genetic analyses were constrained by the low abundances found in the field (Supplementary Table 7).

The understanding of trophic flows within aquatic biota is fundamental to deciphering biochemical fluxes, but very few studies have attempted to fill this knowledge gap in subterranean environments^70^. Some studies have attempted to model groundwater ecosystem ecological functioning^76,77^, but none of them has focused on wide multidisciplinary designs. Here we combined information from previous food web investigation on stygofauna^7,78^ and microbial patterns^22^ with the information gathered via isotopic (carbon and nitrogen) and genetic data on stygofaunal gut biomes from this study (Fig. 5). High rainfall events (see Supplementary Fig. 1 for rainfall categorisation) trigger ecological shifts characterised by a tendency towards more deterministic interactions. Bottom-up controlled microbial communities are proposed as major drivers regulating the trophic trajectories of stygofaunal specimens. The suggested modelling infers selective biofilm proliferation as a driver for increased biological activities in grazers (copepods and amphipods), which are the ultimate target of top predators (beetles, larvae and adults). Given the urgent need to widen the current knowledge of groundwater ecology trends, this investigation provides novel modelling that can bring further light to the processes regulating biodiversity in groundwater ecosystems. The understanding of these dynamics is crucial to evaluate their current conservation status and investigate future trends both in pristine and contaminated groundwaters.

## Supplementary Information

Supplementary Information.
