# Supplementary material for: Rainfall as a trigger of ecological cascade effects in an Australian groundwater ecosystem
Source: Sci Rep. 2021 Feb 12;11:3694. doi: 10.1038/s41598-021-83286-x (PMC7881013; doi:10.1038/s41598-021-83286-x)
Supplement: Supplementary file 1 — Supplementary Information. [file 41598_2021_83286_MOESM1_ESM.docx]

**Rainfall as a trigger of ecological cascade effects in an Australian groundwater ecosystem**

Mattia Saccò, Alison J. Blyth, William F. Humphreys, Steven J.B. Cooper, Nicole E. White, Matthew Campbell, Mahsa Mousavi-Derazmahalleh, Quan Hua, Debashish Mazumder, Colin Smith, Christian Griebler, Kliti Grice

**Supplementary material**


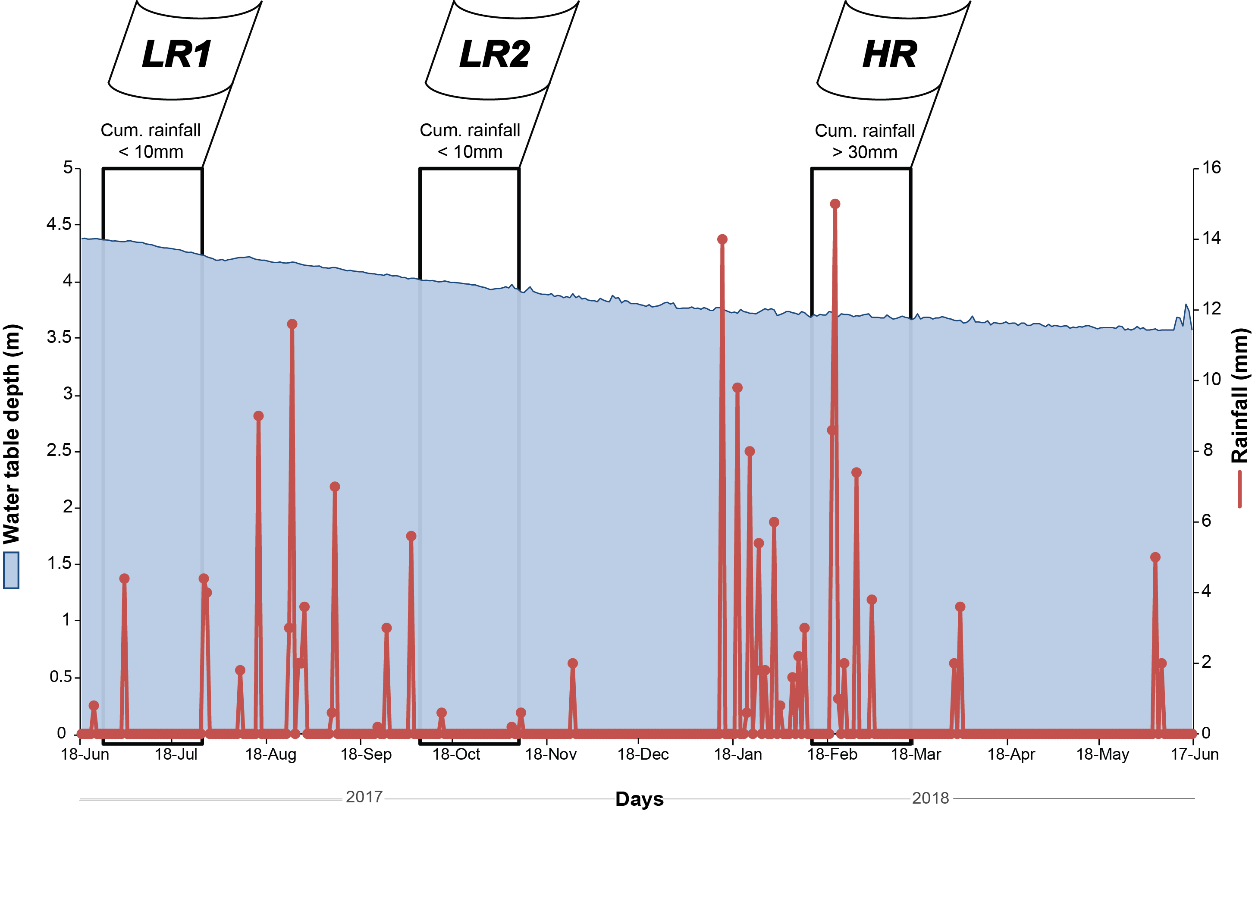


**Supplementary Fig. 1 |** **Weather station data from bore E7 adapted from Saccò *et al.* (23)**. Water level (in mm) are illustrated in light blue and rainfall events (in mm) are shown in red. Flags indicate the dates of LR1 (26/07/2017), LR2 (07/11/2017) and HR (17/03/2018) campaigns, and the black rectangles illustrate the cumulative rainfall and water depth trend for the 30 days before samplings (for rainfall periods category thresholds refer to Hyde *et al.* (53).


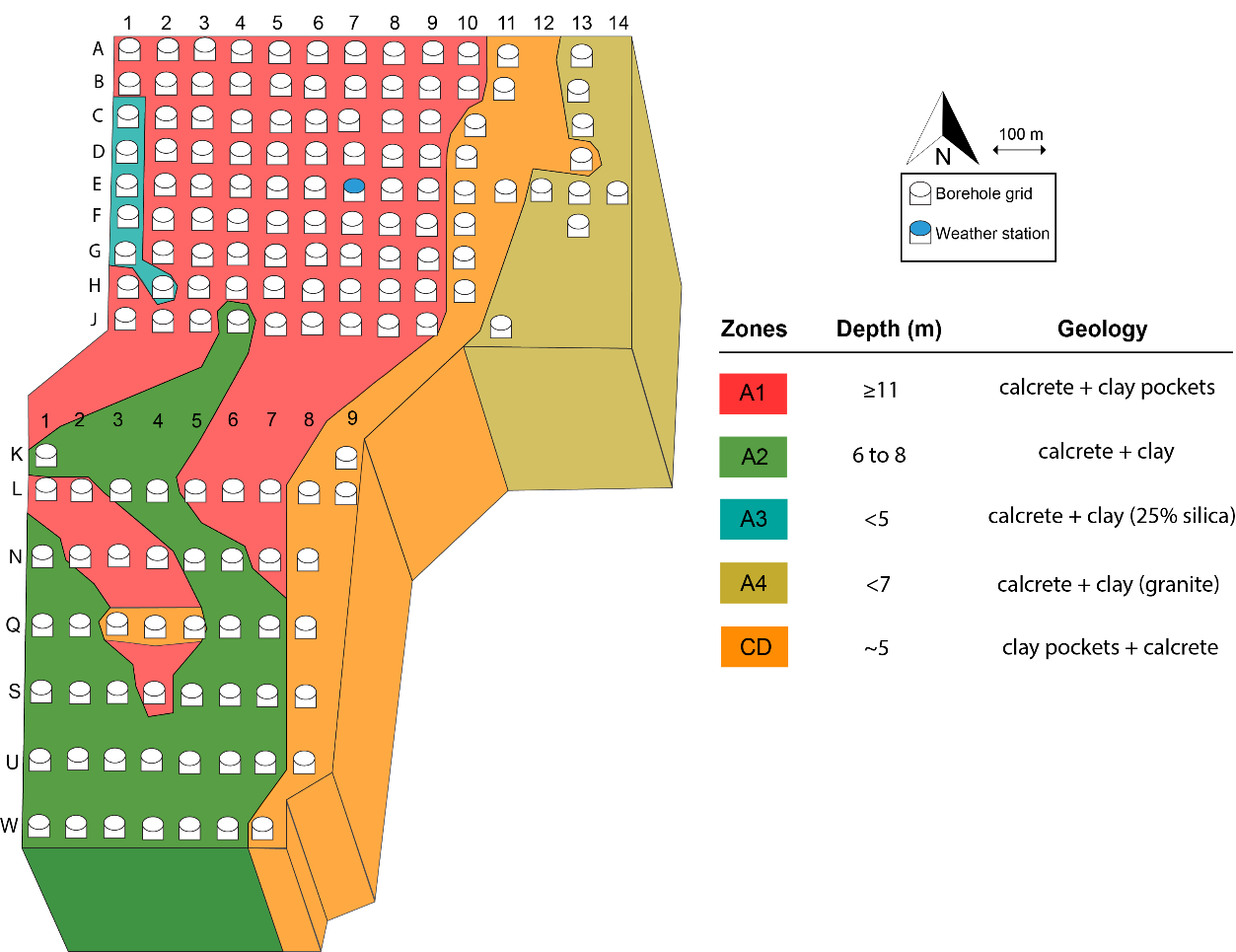


**Supplementary Fig. 2 |. Sturt Meadows geological zones and location of the weather station (bore E7) adapted from Saccò *et al*. (23).**


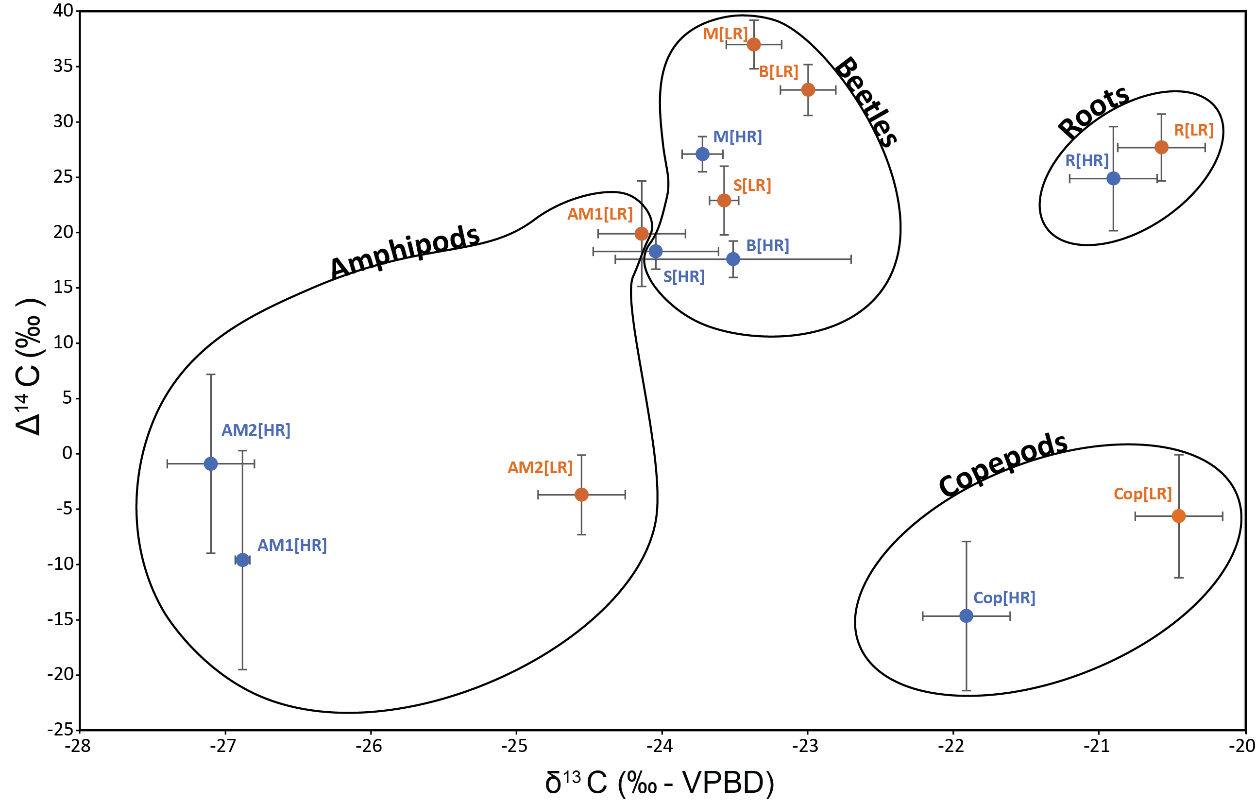


**Supplementary Fig. 3 |** **Biplot illustrating δ^13^C and Δ^14^C data from roots, beetles (B, M and S), amphipods (AM1 and AM2) and copepods (Cop, as a compendium of cyclopoids and harpacticoids).** Refer to Table 1 and Supplementary Table 4 for the specific isotopic values of each group.


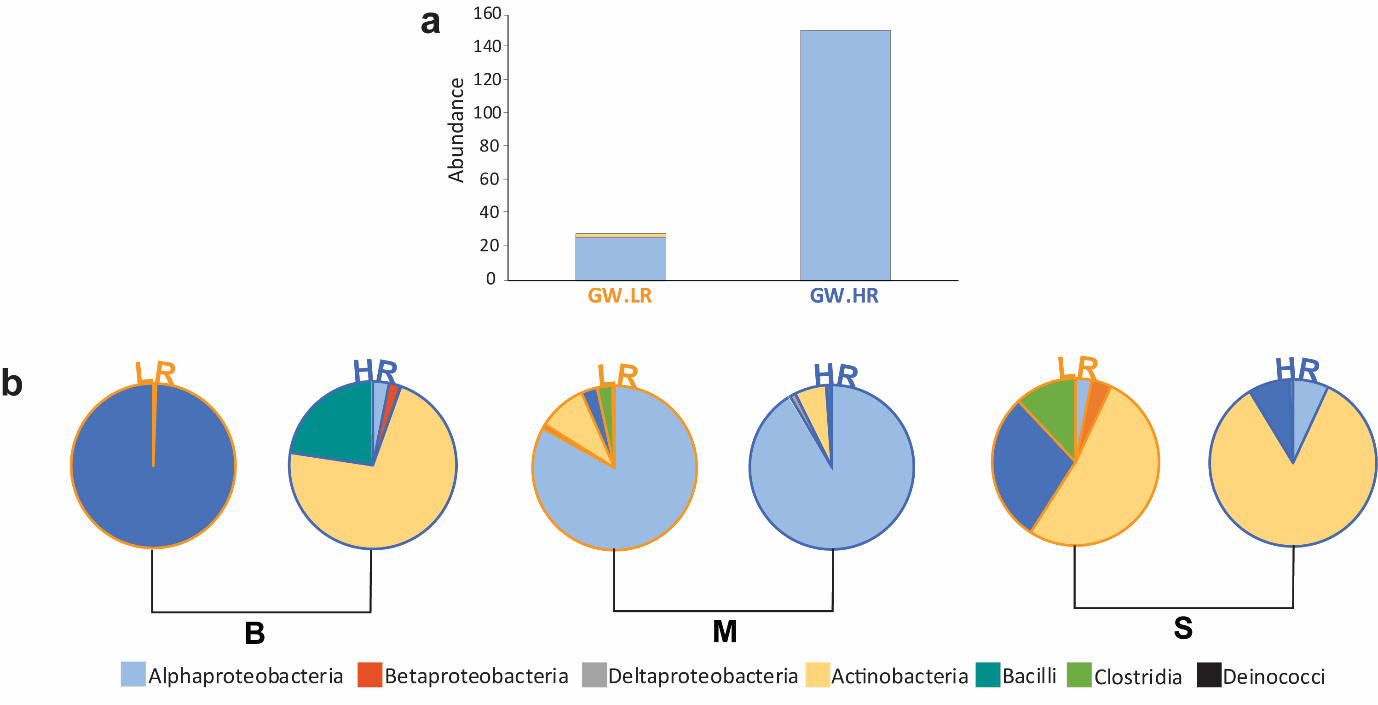


**Supplementary Fig. 4 |Bar plots (a) illustrating the bacterial abundances (corresponding to 37 ZOTUs) in groundwater samples (see Saccò *et al.* (22) for further details) under LR (GW.LR, in orange) and HR (GW.HR, in light blue) and pie charts (b) showing the relative abundances (in %) of the bacterial classes in beetles B, M and S under LR and HR.**


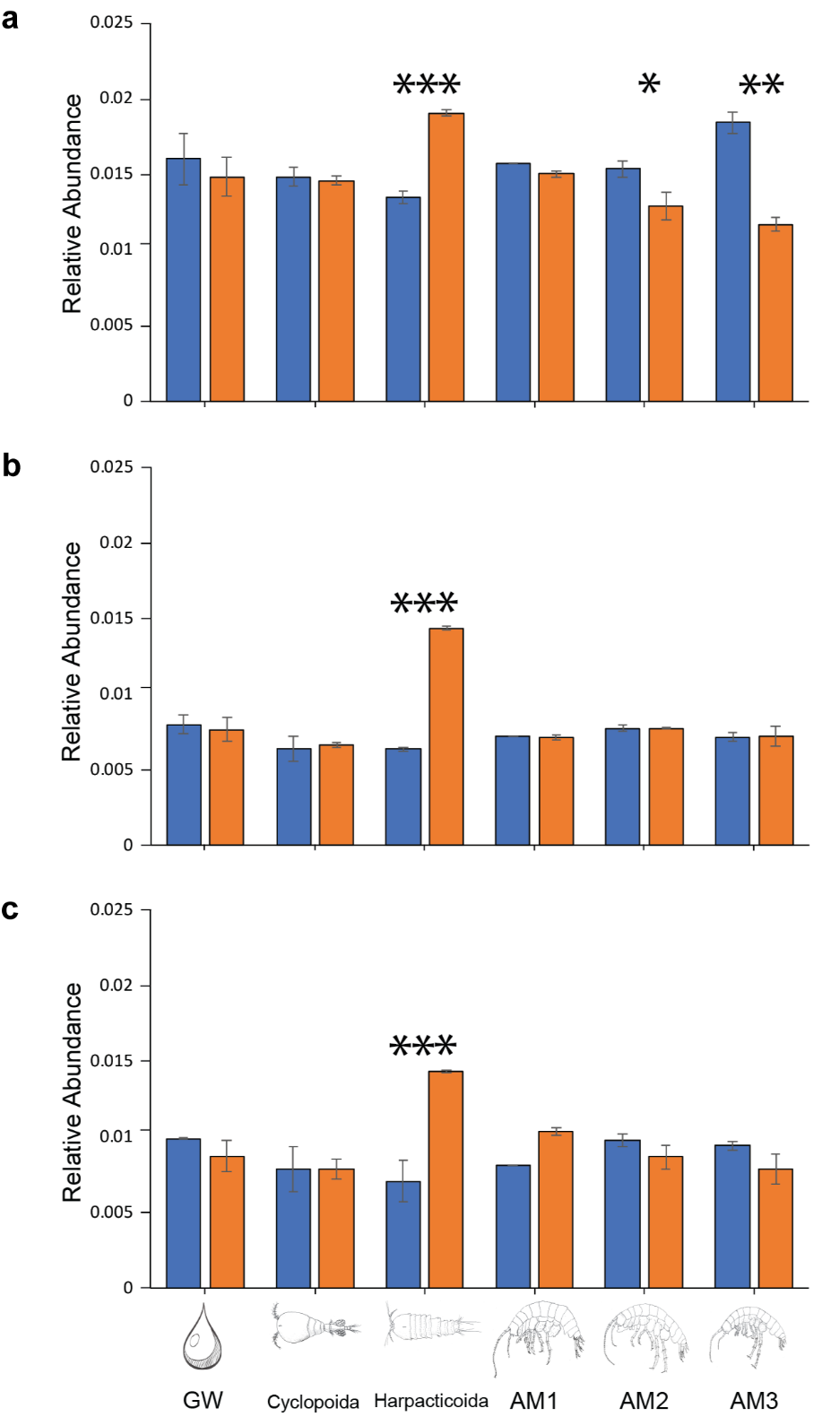


**Supplementary Fig. 5 |** **Abundances of the major KEGG pathways associated with methane metabolism (a), nitrogen metabolism (b) and sulfur metabolism (c).** *, P < 0.05; **, P < 0.005; ***, P < 0.0005.

**Supplementary Table 1** **|** **ANOVAs on recharges and taxonomic groups/roots with Tukey’s pairwise comparisons between the values of Phenylalanine (Phe), Arginine (Arg) and Valine (Val) per each sample.** In bold the non-significative results.

**Supplementary Table 2 | Tukey’s pairwise comparisons of δ^13^C_Val-Phe_ values of the groups pertaining to LR.** *, P < 0.05; **, P < 0.005; ***, P < 0.0005.

**Supplementary Table 3 | Tukey’s pairwise comparisons of δ^13^C_Val-Phe_ values of the groups pertaining to HR.** *, P < 0.05; **, P < 0.005; ***, P < 0.0005.

**Supplementary Table 4 |** **Results from the δ^13^C, δ^15^N and ^14^C analyses on beetles (B, M and S) during LR and HR.** BP: before present.

**Supplementary Table 5 |** **Bacterial abundances in groundwater samples (GW) and samples of amphipods AM1, AM2 and AM3 during LR and HR**. Information extracted from the abundances of ZOTUs per each class.

**Supplementary Table 6 |** **Dietary contributions (in %) of copepods (cyclopoida and harpacticoida) and amphipods AM1 and AM2 under LR and HR.**

**Supplementary Table 7 |** **Abundance data (adapted from Saccò *et al*. (23)) of the different stygofaunal taxa at Sturt Meadows detected during the sampling campaigns LR1, LR2 and HR (first day of sample collecting).**


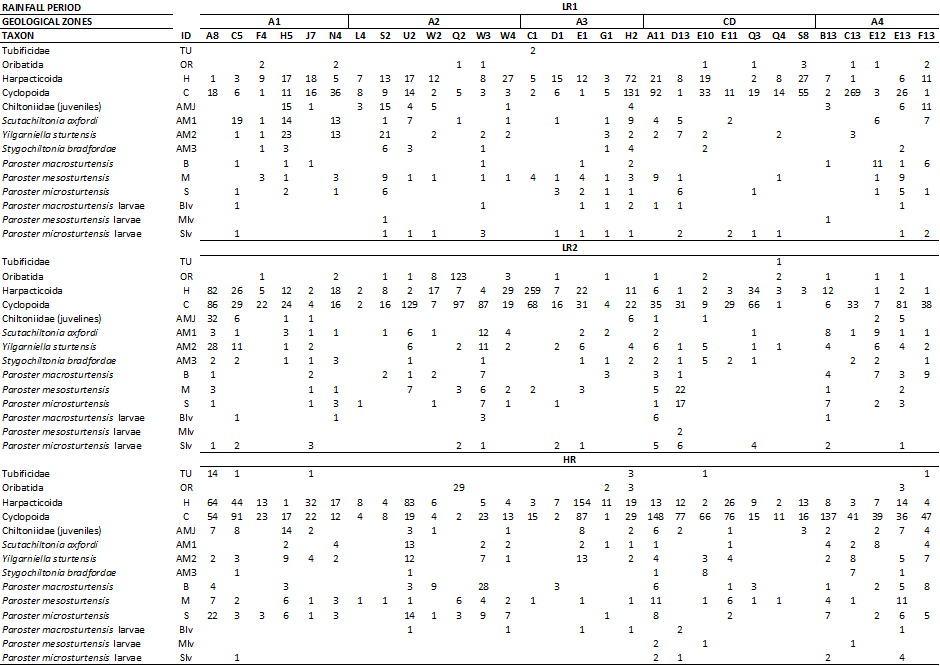


**Supplementary Table 8 |** **Values (mean** **±SD) of the *in situ* hydrochemical parameters. PSS, Practical Salinity Scale; DO, Dissolved Oxygen; ORP, oxidation reduction**
